# MAP4 phosphorylation induced by ARID1A loss sensitizes colorectal cancer cells to EMP

**DOI:** 10.1038/s41419-025-08286-5

**Published:** 2025-12-08

**Authors:** Lei Pan, Danzhu Wu, Yilin He, Kejin Wang, Yingyi Zeng, Cheng Xiang, Lifang Huang, Wenjie Qin, Xu Zhang, Zihuan Wang, Yingnan Yu, Zhen Wang, Li Xiang, Changjie Wu, Aimin Li

**Affiliations:** 1 Guangdong Provincial Key Laboratory of Gastroenterology, Department of Gastroenterology, Nanfang Hospital, Southern Medical University, Guangzhou, China; 2 Department of Gastroenterology, The Second Affiliated Hospital, School of Medicine, The Chinese University of Hong Kong Shenzhen & Longgang District People’s Hospital of Shenzhen, Shenzhen, China; 3 Department of Gastroenterology, Zhuhai People’s Hospital (Zhuhai Hospital Affiliated with Jinan University), Zhuhai, China

**Keywords:** Translational research, Drug development, Targeted therapies, Colon cancer

## Abstract

Mutational inactivation of the tumor suppressor gene *ARID1A* is a key driver of tumorigenesis in various types of cancer, making it a promising therapeutic target for anticancer drug development. Here, we performed a synthetic lethal drug screening in an approved drug library with ARID1A isogenic CRC cell lines and identified estramustine phosphate sodium (EMP), an FDA approved antimicrotubule chemotherapy drug, as a synthetic lethal partner of ARID1A. ARID1A loss increases the vulnerability to EMP. Mechanistically, ARID1A loss increases the phosphorylation level of MAP4 (microtubule-associated protein 4), which is a key microtubule dynamics regulator in cancer cells. Therefore, ARID1A loss attenuates microtubule stabilizing activity of MAP4 and creates a dependence on its residual activity. By targeting MAP4, EMP severely disrupts microtubule dynamics, affecting bipolar spindle formation and positioning, and inducing mitotic cell death in ARID1A-deficient cells. Furthermore, we identified that MAP4 is phosphorylated by PI3K, which is activated by ARID1A loss. These findings highlight MAP4 as a key regulator of microtubule dynamics in ARID1A-deficient cells and unveil a novel synthetic lethality relationship between ARID1A and EMP.

## Introduction

The tumor suppressor AT-rich interactive domain 1A (ARID1A) is a subunit of the SWI/SNF complexes, which remodels chromatin structure to regulate transcriptional activity by binding to transcription factors or transcriptional coactivator/corepressor complexes [1]. Mutational inactivation of ARID1A is found in various type of cancers, and its loss leads to several tumorigenic consequences, including enhanced proliferation and resistance to apoptosis [2]. In colorectal cancer (CRC) patients, mutations in the ARID1A gene occur in approximately 10% of cases [3]. However, immunohistochemical analyses reveal a more substantial impact at the protein level: 25.8% of primary CRC tumors lacked ARID1A expression, and 51.2% showed low expression, resulting in 77% of all CRC samples exhibiting absent or reduced ARID1A levels [4]. Mutational inactivation of ARID1A leads to dysregulation in the PI3K/AKT pathway, WNT pathway, DNA damage response, and tumor immune microenvironment [5–7], suggesting ARID1A is a potential therapeutic target in CRC.

CRC is one of the most lethal malignancies, ranking third in global incidence and second in cause of cancer mortality [8]. Although the treatment methods for CRC have achieved continuous improvement, a proportion of patients remain refractory to the treatments [9, 10]. Therefore, novel therapeutic strategies are urgently needed. In recent years, synthetic lethality has arisen as an attractive therapeutic strategy for the treatment of cancer, because it can be exploited to selectively target cancer cells while sparing normal, healthy cells. Tumor suppressor genes were usually subjected to inactivation mutation, making them difficult to target directly. However, synthetic lethality provides a new insight for tackling targets that are classically undruggable. PARP inhibitors, which were developed based on the principle of synthetic lethality, have been approved for the treatment of ovarian cancer and breast cancer carrying *BRCA* mutation [11]. Thereafter, a variety of synthetic lethal drugs have developed successively, and some of them have progressed to clinical phases [12]. These illustrate that the discovery of synthetic lethal interactions facilitates the indirect targeting of tumor suppressor genes, which might be undruggable. Based on the strategy of synthetic lethality and drug repurposing, we conducted a drug repurposing screening in an approved drug library in *ARID1A* isogenic CRC cells and identified estramustine phosphate sodium (EMP) as a potent synthetic lethal candidate for ARID1A-deficient CRC cells.

EMP, an estradiol analog, functions as an antimicrotubule chemotherapy agent. It is approved for the palliative management of metastatic and/or progressive prostate carcinoma and widely available across the United States, Japan, and many countries throughout Europe and the Middle East. It depolymerizes microtubules by binding to microtubule-associated proteins (MAPs), resulting in G2/M arrest and apoptosis in cells [13, 14]. MAPs, defined as proteins, which interact with microtubules, play a key role in microtubule stability. MAPs can decorate the microtubules lattice and stabilize them by increasing the rate of polymerization and inhibiting the rate of depolymerization, and promote growth and reduce shrinkage speeds, enhancing the stability of microtubules [15]. In mammals, MAPs family mainly includes MAP1A, MAP1B, MAP2, MAP4, Tau, and so on. Basing on their cellular expression patterns, MAP1A, MAP1B, MAP2, and Tau are expressed predominantly in neuronal cells, while MAP4 is mainly in nonneuronal cells and ubiquitously found in all cell types [16]. As a key regulator of microtubule stability, MAP4 is considered as an important effector in a variety of cancers. MAP4 activity is regulated by its phosphorylation level. Phosphorylation of MAP4 leads to its detachment from microtubules, thus disrupting this stabilization [15]. In mitosis, phosphorylation of MAP4 reduces its affinity to microtubules, which affects the movement of chromosomes.

In the present study, we aimed to search for synthetic lethal drug for ARID1A-deficient CRC in an approved drug library and explore the underlying molecular mechanism. We found that EMP treatment or MAP4 silencing selectively induces apoptosis in ARID1A-deficient cells. Our mechanistic exploration of the synthetic lethality between EMP and ARID1A suggests that PI3K protein is notably activated in ARID1A-deficent cells where it binds to MAP4 and facilitates its phosphorylation, thereby increasing the sensitivity of ARID1A-deficient CRC cells to EMP.

## Materials and methods

### Cell culture and reagents

HCT116 *ARID1A* isogenic cell lines and RKO cells were kindly gifted from Prof. Joong Sup Shim (University of Macau, Macau, China). HCT116 *ARID1A* isogenic cell lines and RKO *ARID1A* isogenic cell lines were cultured in RPMI-1640 and DMEM medium containing 10% FBS, respectively. All cells were cultured at 37 °C in an incubator containing 5% CO_2_. EMP (T4451), vinorelbine (T0190), and paclitaxel (T0968), LY294002 (T2008), GSK2334470 (T2348), MK2206 (T1952), nocodazole (T2802) and colchicine (T0320) were purchased from TargetMol (USA).

### Generation of RKO ARID1A overexpression cell lines

pLenti-puro-ARID1A (Addgene plasmid #39478) was kindly gifted from Prof. Joong Sup Shim (University of Macau, Macau, China). RKO cells were transfected with ARID1A overexpression plasmids using Lipofectamine 3000 (Invitrogen), and the ARID1A overexpression clones were screened with 1 μg/ml puromycin. The overexpression efficiency of ARID1A were verified by western blotting.

### Approved drug library screening

An approved drug library (L1000) containing 2342 drugs was purchased from TargetMol (USA). Each compound was diluted with PBS and arrayed in 384-well plates at a final concentration of 200 μM. HCT116 and HCT116 *ARID1A*-KO #2 cells were screened with the approved drug library at a final concentration of 20 μM. After 72 h-drug incubation, cell viability was then measured using AlamarBlue reagent. The fluorescence intensity (excitation 560 nm, emission 590 nm) was detected using SpectraMax-M4 (Molecular Devices, Sunnyvale, CA). Cell viability of each compound was calculated using GraphPad Prism 8 software.

### Cell cycle and apoptosis assays

Cell cycle and apoptosis assays were performed according to the manufacturer’s instructions. Briefly, cells treated with EMP were harvested, washed with PBS. For cell cycle analysis, cells were resuspended in 1 ml DNA staining solution containing 10 μl permeabilization solution for 30 min. For apoptosis analysis, cells were resuspended in 500 μl binding buffer containing 5 μl Annexin V-FITC and 5 μl DAPI at room temperature for 5 min. Data were acquired and analyzed using BD Accuri C6 Software (1.0.264.21) and FlowJo v10 software (FlowJo LLC, USA), respectively.

### Western blot and antibodies

Cells or tissues were lysed using RIPA buffer, and total proteins were harvested. Equal amounts of protein were subjected to SDS-polyacrylamide gel electrophoresis and transferred onto PVDF membranes. The membranes were subsequently blocked with 5% skimmed milk for 2 h and incubated with primary antibodies, including ARID1A (Cell Signaling Technology, #12354S), MAP4 (Proteintech, 11229-1-AP), Phospho-MAP4 (Ser696) (Invitrogen, PA5-105813), PI3K (Cell Signaling Technology, #4249S), PDK1 (Abmart, T56926F), AKT (Abmart, T55561F), Phospho-AKT(Thr308) (Cell Signaling Technology, #9275T), Phospho-AKT(Ser473) (Cell Signaling Technology, #4060T), α-tubulin (Abmart, P60049S), Cleaved-PARP (Cell Signaling Technology, #5625T), Cleaved-Caspase3 (Cell Signaling technology, #9664T) and GAPDH (Fdbio science, FD0063) antibodies, followed by incubation with horseradish peroxidase (HRP)-labeled secondary antibodies.

### Tumor xenograft mouse model

All animal procedures were approved by the Animal Experimental Ethics Committee of Southern Medical University (Ethical approval number: IACUC-LAC-20220715-001). Four-week-old female nude mice were injected subcutaneously into both flank with 100 μl mixture containing 2 × 10^6^ HCT116 *ARID1A*^+/+^ (left flank) and HCT116 *ARID1A*^−/−^ (right flank) cells in 50% PBS and 50% Matrigel. Once tumors were palpable, mice were randomized into two groups for treatment with vehicle or EMP, with 5 mice in each group. Mice were treated with vehicle (sterile saline, containing 5% DMSO, 5% tween-80 and 5% polyethylene glycol-400, daily) and EMP (20 mg kg^−1^, daily) for 17 days via i.p. injection. Tumor size was measured every two days, and tumor volume was determined by the modified ellipsoid formula (long axis × short axis^2^ × π/6). Periodical measurements of the mouse body weight were taken to assess potential drug toxicity. At the end of the study, mice were sacrificed, and tumors were dissected and weighed.

### Microtubule polymerization assay

Polymeric and soluble fractions of tubulin were extracted from cultured cells based on the previous report [17]. Briefly, after 48 h drug treatment or siRNA transfection, cells were harvested, washed once with PBS, and then resuspended in extraction buffer, containing 0.1 M PIPES, pH 7.1, 1 mM MgSO_4_, 1 mM EGTA, 2 M glycerol, 0.1% TritonX-100, and protease inhibitors. Cells were lysed on ice for 15 min and centrifuged at 21,000 × *g* for 15 min at 4 °C. The supernatant containing the soluble fraction of tubulin was collected. The pellet was resuspended in lysis buffer (containing 25 mM Tris-HCl, pH 7.4, 0.4 M NaCl and 0.5% SDS) and boiled for 10 min, followed by centrifugation at 21,000 × *g* for 5 min and collection of polymeric tubulin. The polymeric (P) and soluble (S) fractions of tubulin were subjected to Western blot analysis. Anti-GAPDH was used as an internal control. The relative microtubule polymerization status was estimated by dividing polymeric tubulin by total tubulin (polymeric plus soluble).

### Immunofluorescence analyses of microtubule polymerization, spindle abnormalities, and mitotic defects

For the analyses of microtubule polymerization, HCT116 *ARID1A* isogenic cell lines were seeded on coverslips and treated with EMP or VNR for 48 h. For the analyses of spindle abnormalities and mitotic defects, HCT116 *ARID1A* isogenic cell lines were treated with EMP or microtubule antagonists for 48 h. Prior to 4% paraformaldehyde fixation, cells were treated with bortezomib (100 nM) for 2 h to arrest mitotic cells at the metaphase-to-anaphase transition [17]. After permeabilization with 0.5% TritonX-100 and blocking with 5% BSA, the fixed cells were incubated with primary antibodies against α-tubulin (Abmart, P60049S) or Tyr-tubulin (Sigma-Aldrich, T9028) overnight at 4 °C. The following day, anti-rabbit Alexa Fluor 488 or anti-mouse Alexa Fluor 594 secondary antibodies were incubated for 1 h at room temperature. Actin was stained by incubation with 647-labeled Phalloidin (YEASEN, 40762ES75) for 1 h, and the nuclei were stained with DAPI. Images were acquired using the Carl Zeiss LSM880 system in conjunction with Airyscan or fluorescence microscope (Leica THUNDER DMi8). The microtubule polymerization index was calculated according to the method described by Harkcom et al. [18]. The total cell area was determined as the area of phalloidin staining. The microtubule polymerization index was defined as the ratio of polymerized microtubules to cell area. For the analyses of spindle abnormalities, spindle length was measured using Image J software, and abnormal spindle morphology and mitotic defects were recorded based on previous report [19, 20].

### Co-immunoprecipitation

HCT116, RKO, and 293T cells were harvested using RIPA buffer. Protein lysates were precleared by rotary incubation with 500 μl protein A/G magnetic beads containing rabbit normal IgG at 4 °C for 1 h. Then the supernatant was collected by magnetic separation followed by rotary incubation with protein A/G beads and MAP4 or PI3K antibodies at 4 °C overnight. The following day, protein A/G magnetic beads-antibody-interacting protein compounds were separated from the protein lysates and washed gently three times with 1 ml RIPA buffer containing protease inhibitor. Finally, the compounds were resuspended in 1× loading buffer and subjected to Western blot analyses.

### Statistical analyses

All data were analyzed using SPSS version 20.0 (SPSS Inc., Chicago, IL) and presented as mean ± SD or SEM. Two-sided Student’s *t* test was used to analyze variables between the control and test groups. Statistical significance for all tests was set at *P* value <0.05.

## Results

### Approved drug library screening identifies EMP as a synthetic lethal drug for ARID1A loss

**Fig. 1:**
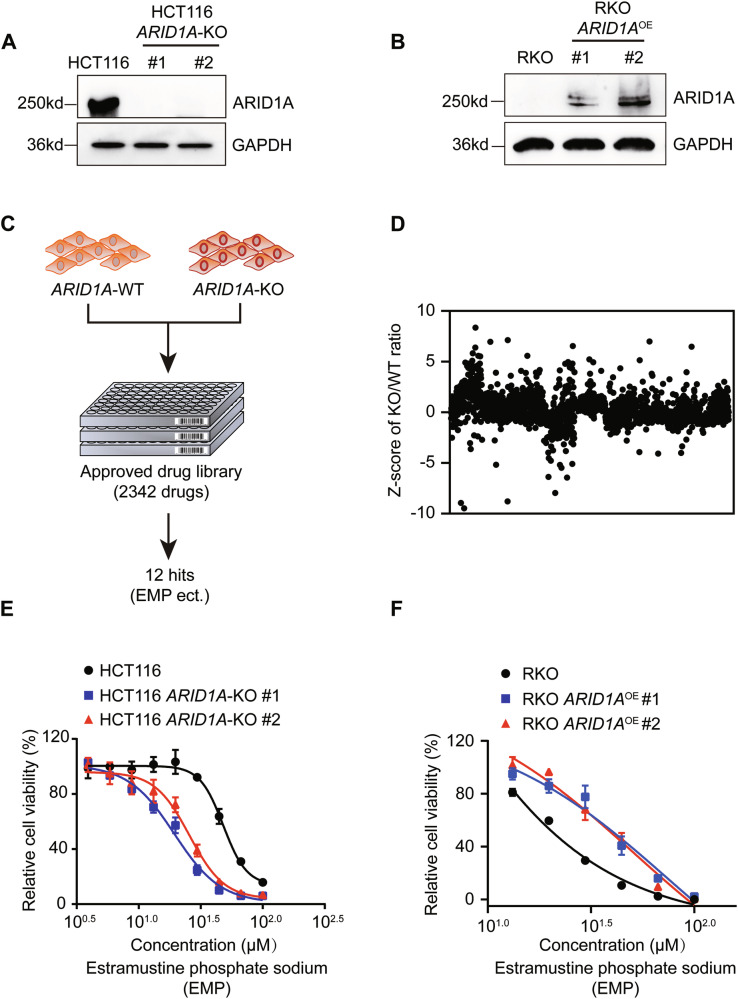
Identification of EMP as a synthetic lethal partner of ARID1A in CRC cells. **A**, **B** *ARID1A* knockout and overexpression efficiency in HCT116 and RKO cell lines. **C** A flowchart of synthetic lethality screening procedure. **D** Scatter plot of the Z-scores for approved drug library screening. Dose-response curves of HCT116 (**E**) and RKO (**F**) *ARID1A* isogenic cell lines treated with EMP. The data are presented as mean ± SD, n = 3.

To screen and verify candidates that might show synthetic lethal effects with ARID1A, *ARID1A* knockout and overexpression cell lines were constructed. HCT116 *ARID1A* knockout cell lines were generated previously by our group [21], and ARID1A status was verified by western blotting (Fig. 1A). RKO cells harbor an *ARID1A* frameshift deletion mutation [22]. Therefore, we reintroduced *ARID1A* in RKO cells via the ARID1A plasmid, and the expression levels were determined by western blotting (Fig. 1B). To investigate potential synthetic lethal effects based on *ARID1A* status, we screened an Approved Drug Library containing 2342 drugs approved by the Food and Drug Administration (FDA), the European Medicine Agency (EMA), and the National Medical Products Administration (NMPA) using HCT116 *ARID1A* isogenic cell lines. The screening was conducted at a concentration of 20 µM, and estramustine phosphate sodium (EMP), pyrvinium pamoate, lumacaftor, and cisplatin, et al. exhibited synthetic lethal effects on ARID1A-deficient cells (Fig. 1C, D). To validate the screening results, we conducted a dose-response experiment using HCT116 *ARID1A* isogenic cell lines. EMP and pyrvinium pamoate treatments showed decent selectivity toward two *ARID1A*-KO clones compared to wild-type HCT116 cells (Figs. 1E and S1A), but the phenotypic effects of the other hits were limited (Fig. S1B–K). Considering the relationship between ARID1A, pyrvinium pamoate and the WNT pathway has been intensively investigated [23, 24], we selected EMP as the primary synthetic lethal compound for follow-up studies. Our results showed that the IC50 values for EMP in HCT116 *ARID1A*-WT cells and two *ARID1A*-KO cells were 44.50, 19.89, and 29.38 µM, respectively, representing 2.24-fold and 1.51-fold changes in the knockout cells compared to the wild-type cells (Fig. 1E). In RKO *ARID1A* isogenic cells, the IC50 in RKO ARID1A-deficient cells was 19.95 µM, whereas in RKO *ARID1A*-expressing cells, the IC50 values were 44.26 and 44.37 µM, indicating 2.22-fold changes in the reintroduced ARID1A cell lines (Fig. 1F). These results demonstrate that EMP exhibits consistent selective vulnerability in ARID1A-deficient CRC cells. In this work, we revealed a novel synthetic lethal effect between ARID1A and EMP, presenting promising translational implications.

### EMP exhibits synthetic lethal effects with ARID1A loss in vitro and in vivo

**Fig. 2:**
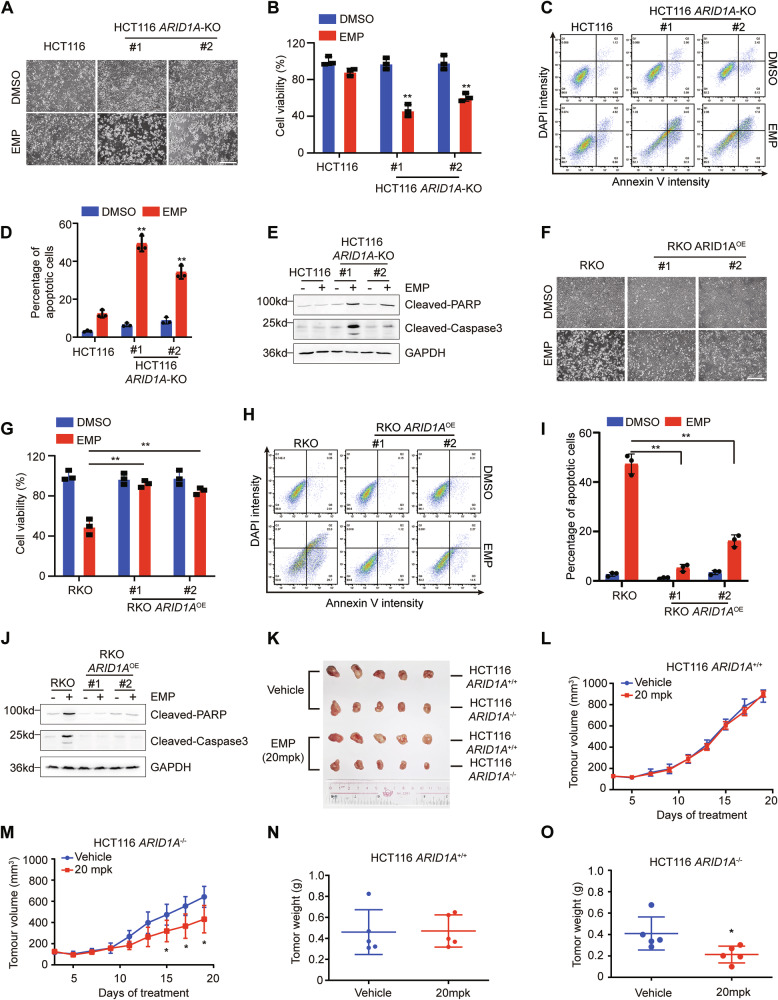
In vitro and in vivo synthetic lethality between EMP and ARID1A in CRC cells. Representative images (**A**) and statistical analysis (**B**) of cell viability are shown. Scale bar, 200 μm, n = 3. **C**, **D** EMP (15 μM) selectively induced apoptosis in ARID1A-deficient CRC cells, n = 3. **E** Apoptosis-related proteins were determined by Western blotting. Representative images (**F**) and cell viability (**G**) of RKO *ARID1A* isogenic cell lines treated with EMP (20 μM) are shown. Scale bar, 200 μm, n = 3. **H**, **I** Re-introduction of ARID1A in RKO cells reversed EMP-induced apoptosis, n = 3. **J** Apoptosis-related proteins were examined by Western blotting. **K** Schematic representation of the mouse tumor xenograft model. Tumor growth curve (**L**, **M**) and tumor weight (**N**, **O**) of HCT116 *ARID1A*^+/+^ and HCT116 *ARID1A*^−/−^ xenografts treated with vehicle or 20 mg/kg EMP are presented, n = 5. In all relevant panels, data are presented as mean ± SD, ^*^P < 0.05, ^**^P < 0.01, Student’s *t* test.

To further investigate the synthetic lethality between ARID1A and EMP, cell viability and apoptosis were examined. Compared with HCT116 cells, EMP treatment selectively inhibited viability and induced apoptosis in ARID1A-deficient cells (Fig. 2A–E). In addition, the colony formation assay showed that EMP potently reduced clonogenic survival (Fig. S2A, B). Similar results were observed in RKO *ARID1A* isogenic cells. Re-introduction of ARID1A in *ARID1A*-mutant RKO cells significantly reversed EMP-induced cell viability decreasing, growth inhibition and apoptosis (Figs. 2F–J and S2C, D). Above results indicate that EMP exerts a synthetic lethal effect on ARID1A-deficient cells.

Next, we validated the synthetic lethality of EMP and ARID1A in vivo. A xenograft mouse model was established using HCT116 *ARID1A* isogenic cell pair (Figs. 2K and S2E). EMP treatment showed negligible suppressive effects on HCT116 *ARID1A*^+/+^ tumor xenografts (Fig. 2L, N), whereas it significantly inhibited HCT116 *ARID1A*^−/−^ xenograft tumor growth (Fig. 2M, O). We further analyzed tumor samples isolated from mice and observed that EMP treatment selectively inhibited tumor cell proliferation and induced apoptosis in HCT116 *ARID1A*^−/−^ xenograft mice (Fig. S2F, G). Meanwhile, the body weight of mice in the vehicle and EMP treatment groups showed no obvious changes during the treatment period, indicating that EMP did not appear to cause a toxic effect on mice (Fig. S2H). In summary, these results collectively demonstrate that EMP treatment induces synthetic lethality in ARID1A-deficient CRC cells, both in vitro and in vivo.

### ARID1A loss sensitizes CRC cells to EMP-induced disruption of microtubule dynamics

**Fig. 3:**
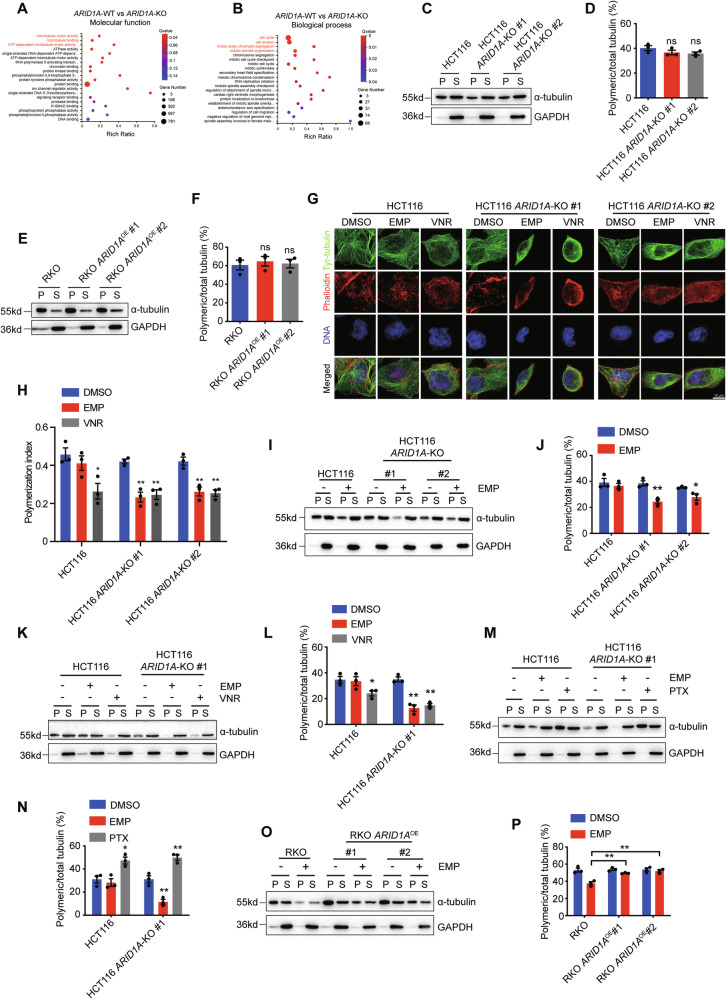
EMP destabilizes interphase microtubule dynamics of ARID1A-deficient CRC cells. **A**, **B** Gene ontology (GO) enrichment analyses of transcriptome sequencing. Analyses of microtubule polymerization in HCT116 (**C**, **D**) and RKO (**E**, **F**) *ARID1A* isogenic cell lines, n = 3. Representative images of immunofluorescence staining (**G**) and analysis of microtubule polymerization index (**H**), Scale bar, 10 μm, n = 3. **I**–**N** Effect of EMP on the microtubule polymerization. Microtubule polymerization assay was performed to detect the expression levels of polymeric and soluble tubulin (**I**, **K**, **M**), and quantification of polymeric microtubules are shown (**J, L, N**). VNR (6 nM) and PTX (6 nM) were used as positive control, n = 3. Effect of EMP on the microtubule polymerization of RKO *ARID1A* isogenic cell lines. Microtubule polymerization (**O**) and quantification of polymeric microtubules (**P**) are shown, n = 3. In all relevant panels, data are presented as mean ± SEM. ns not significant, ^*^P < 0.05, ^**^P < 0.01, Student’s *t* test. P and S represent polymeric and soluble fractions of tubulin, respectively.

EMP mainly functions as an antimicrotubule drug that disrupts microtubule dynamics by destabilizing the microtubule against polymerization, resulting in G2/M cell cycle arrest [13]. To clarify the potential mechanism underlying the observed synthetic lethality between ARID1A and EMP, we analyzed the transcriptome profile of the HCT116 *ARID1A* isogenic cell pair. Gene ontology (GO) analyses indicated that ARID1A participated in molecular function and biological process related to microtubule motor activity (Fig. 3A) and spindle associated processes, including cell cycle, cell division, mitotic sister chromatid segregation, and mitotic spindle organization (Fig. 3B). To confirm the results obtained from transcriptome sequencing, we assessed the expression levels of the total, polymeric, and soluble fractions of the microtubules. ARID1A loss or overexpression had a negligible influence on the expression level of total α-tubulin protein (Fig. S3A, B), whereas ARID1A loss or overexpression caused a minor decrease or increase in the expression level of the polymeric fraction of microtubules (Fig. 3C–F). These data suggest that ARID1A-deficient cells continuously maintain microtubule dynamics, thus permitting cell survival.

Microtubules are components of the cytoskeleton that function as tracks for intercellular transport and form a framework to position organelles and other cellular components, and regulate cell morphodynamics and physiological process [25]. In mitosis, microtubules are essential for spindle formation and chromosome segregation. As an antimicrotubule agent, EMP disrupts the normal assembly of microtubule required for cell division and proper cell function, leading to cell death eventually. By combining transcriptome profile analyses with the pharmacological effect of EMP on microtubule assembly, we hypothesized that ARID1A-deficient cells were hypersensitive to EMP treatment, which may be due to the synergistic disruption of microtubule dynamics by EMP and ARID1A, ultimately leading to cell apoptosis. To test this hypothesis, polymeric microtubules were visualized by immunofluorescence staining with tyrosinated tubulin (tyr-tubulin) as described by Harkcom et al. [18]. Our results showed that EMP treatment preferentially promoted microtubule depolymerization in ARID1A-deficient cells, and the decrease in the microtubule polymerization index was similar to that of vinorelbine, whereas the polymerization index was slightly reduced in HCT116 cells (Fig. 3G, H). We further verified the results via microtubule polymerization assay. To better observe the alteration of microtubule polymerization status, vinorelbine and paclitaxel were used as positive controls. EMP treatment selectively reduced the polymeric fraction of microtubules in ARID1A-deficient cells, which was comparable to the extent of vinorelbine (Fig. 3I–L), while paclitaxel markedly increased the polymeric form of microtubules (Fig. 3M,N). In RKO *ARID1A* isogenic cells, overexpression of ARID1A reversed the microtubule polymerization status (Fig. 3O, P). The above results illustrate that EMP selectively promotes microtubule depolymerization in ARID1A-deficient cells.

**Fig. 4:**
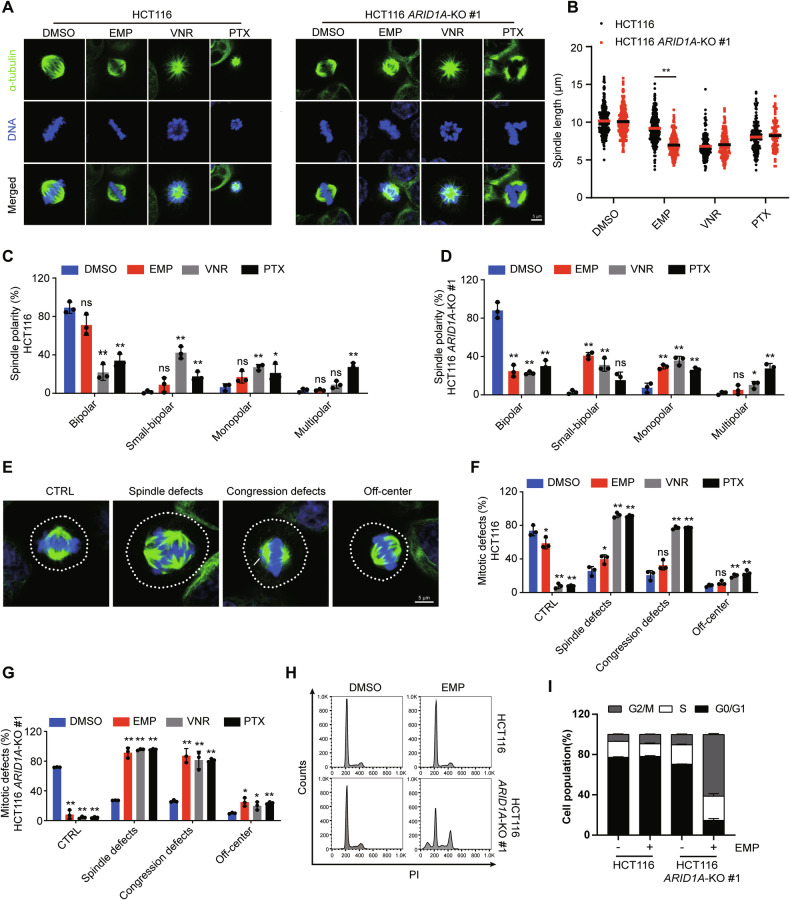
EMP disrupts mitotic spindles of ARID1A-deficient CRC cells. **A** Representative images of spindle abnormalities. Scale bar, 5 μm. **B** Measurement of spindle length. All the bipolar and small-bipolar mitotic spindles were analyzed. **C**, **D** Analyses of abnormal spindle morphologies. All the mitotic spindles were analyzed and classified for quantitative analyses, n = 3. **E** Representative images of mitotic defects. The white dotted lines indicate the cell boundaries, and the white arrow points the lagging chromosomes. Scale bar, 5 μm. **F**, **G** Analyses of mitotic phenotypes. All the metaphase spindles taken from confocal microscope were analyzed. It is notable that multiple mitotic defects phenotypes can co-occur within the same cell, n = 3. Cell cycle distribution (**H**) and cell population quantification (**I**) are shown, n = 3. In all relevant panels, data are presented as mean ± SD. ns not significant; ^*^P < 0.05, ^**^P < 0.01, Student’s *t* test.

The spindle is composed of microtubules that attach to the chromosomes at the kinetochore. The main function of the mitotic spindle is to ensure the proper separation and distribution of chromosomes during cell division, and abnormal spindle formation can cause defects during cell division, including chromosomal instability, structural abnormalities or even cell death [26, 27]. To better characterize the mitotic spindle morphology, we visualized mitotic spindles and chromosomes using immunofluorescence staining. In HCT116 cells, EMP treatment caused spindle shortening but did not significantly affect spindle polarity. Vinorelbine induced significant spindle shortening and a significant increase in small-bipolar and monopolar spindles in HCT116, HCT116 *ARID1A*-KO #1 and HCT116 *ARID1A*-KO #2 cells, whereas paclitaxel induced moderate spindle shortening and a marked increase in monopolar and multipolar spindles. Similar to vinorelbine, EMP induced significant spindle shortening and a significant increase in small-bipolar and monopolar spindles in HCT116 *ARID1A*-KO #1 and HCT116 *ARID1A*-KO #2 cells (Figs. 4A–D andS4A–C). The position of the mitotic spindle is critical for proper cell division, and incorrect placement of the mitotic spindle can result in deleterious consequences, including aneuploidy and cell death [28]. Thus, we examined the effects of EMP on spindle positioning. In both control groups, the mitotic spindles captured and aligned chromosomes on the metaphase plate. In HCT116 cells, EMP treatment increased spindle defects only slightly, while significantly increasing the proportion of mitotic defects, including defects in spindle assembly, chromosome alignment and spindle positioning in ARID1A-deficient cells (Figs. 4E–G and S4D). In line with these observations, EMP selectively induced G2/M arrest in ARID1A-deficient cells (Figs. 4H, I and S4E, F). These data suggest that EMP severely affects spindle assembly, polarity, and positioning in ARID1A-deficient cells.

### The synthetic lethality between ARID1A and EMP is dependent on MAP4 activity

**Fig. 5:**
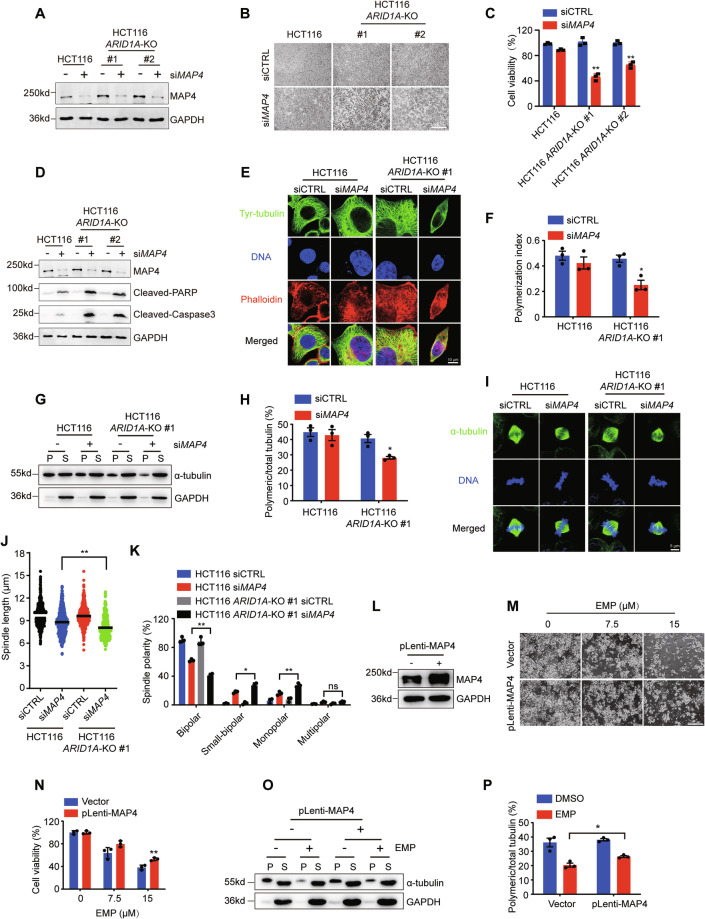
EMP induces synthetic lethality in ARID1A-deficient CRC cells by targeting MAP4. **A** The knockdown efficiency of MAP4 siRNA. Representative images (**B**) and statistical analysis (**C**) of cell viability are shown. Scale bar, 500 μm. **D** Apoptosis-related proteins were detected by western blotting. Representative images of immunofluorescence staining (**E**) and analysis of microtubule polymerization index (**F**), n = 3. Microtubule polymerization assay was performed to detect the expression level of polymeric and soluble tubulin (**G**), and quantification of polymeric microtubules are shown (**H**), n = 3. **I** Representative images of spindle abnormalities. Scale bar, 5 μm. **J** Measurement of spindle length. All the bipolar and small-bipolar mitotic spindles were analyzed. **K** Analyses of abnormal spindle morphologies, n = 3. **L** Ectopic overexpression of MAP4 in HCT116 *ARID1A*-KO #1 cells. Representative images (**M**) and quantification of cell viability (**N**) are shown. Scale bar, 200 μm, n = 3. Microtubule polymerization status (**O**) and quantification of polymeric microtubules (**P**) in HCT116 *ARID1A*-KO #1 cells transfected with MAP4 plasmid and treated with EMP, n = 3. In all relevant panels, data are presented as mean ± SD. ns not significant; ^*^P < 0.05, ^**^P < 0.01, Student’s *t* test. P and S represent polymeric and soluble fractions of tubulin, respectively.

EMP binds to MAP4 and disrupts its activity, leading to the destabilization and depolymerization of microtubules [29]. To address whether the observed synthetic lethal effect between EMP and ARID1A relies on MAP4 level, we silenced MAP4 expression using a specific siRNA and analyzed its synthetic lethal effect in the ARID1A-isogenic cell pair. Similar to EMP treatment, MAP4 silencing preferentially inhibited cell viability and promoted apoptosis in ARID1A-deficient cells (Fig. 5A–D). Microtubule polymerization status was further examined using immunofluorescence staining and microtubule polymerization assay. Our results showed that MAP4 silencing significantly reduced the polymerization index in ARID1A-deficient cells, similar to the phenotype observed in EMP treatment (Fig. 5E–H). In addition, in line with EMP treatment, si*MAP4* selectively caused spindle shortening (Fig. 5I, J), an increased proportion of small-bipolar and monopolar spindles (Fig. 5K), and mitotic defects in spindle assembly, chromosome alignment, and spindle positioning (Fig. S5A, B). To further confirm the targeting of MAP4 by EMP, we ectopically overexpressed MAP4 in ARID1A-deficient cells and examined the cell viability and microtubule polymerization status. Overexpression of MAP4 in ARID1A-deficient cells significantly reversed the inhibition of cell viability and microtubule depolymerization induced by EMP (Fig. 5L–P). Taken together, these results demonstrate that ARID1A-deficient CRC cells are highly dependent on MAP4 activity.

### ARID1A loss-induced MAP4 phosphorylation enhances EMP sensitivity

**Fig. 6:**
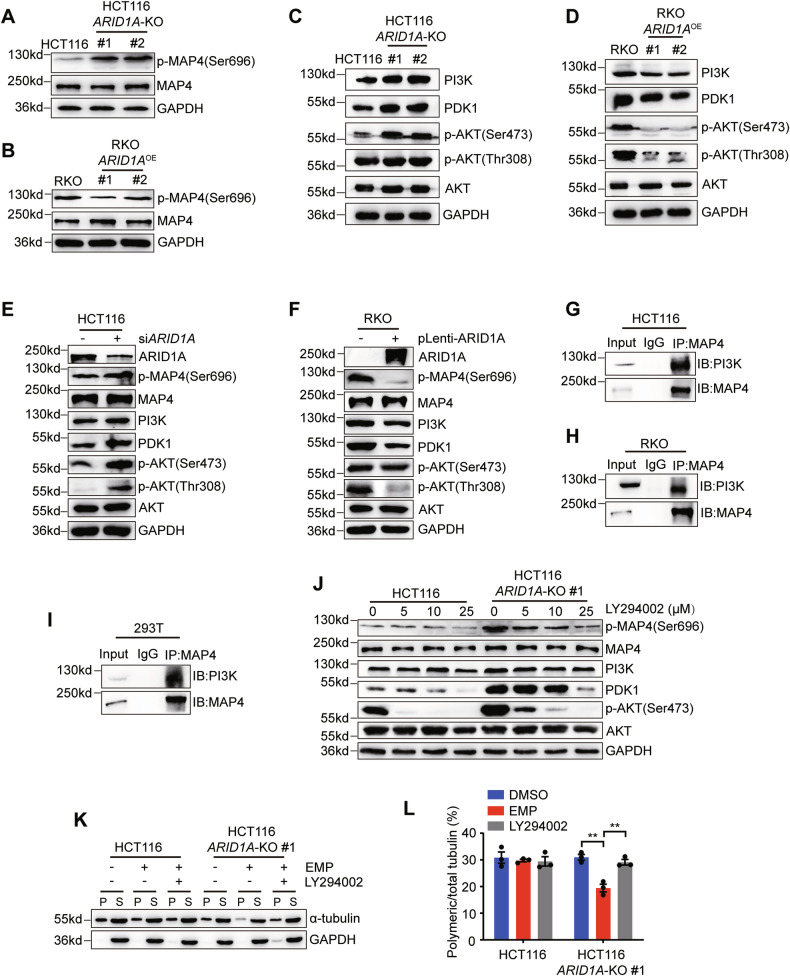
ARID1A loss promotes MAP4 phosphorylation increasing EMP sensitivity. The phosphorylated MAP4 levels in HCT116 (**A**) and RKO (**B**) *ARID1A* isogenic cell lines. Changes in protein expression level of PI3K/AKT signaling pathway in HCT116 (**C**) and RKO (**D**) *ARID1A* isogenic cell lines. Changes in protein expression level of PI3K/AKT signaling pathway induced by ARID1A silencing (**E**) or overexpression (**F**). **G–I** Interaction between MAP4 and PI3K. Co-immunoprecipitation assays were performed in HCT116 (**G**), RKO (**H**) and 293T (**I**) cell lines. **J** Changes of phosphorylated MAP4 expression level in HCT116 *ARID1A* isogenic cell pair treated with LY294002 (PI3K inhibitor). Microtubule polymerization status (**K**) and quantification of polymeric microtubules (**L**) in HCT116 *ARID1A* isogenic cell pair treated with EMP and LY294002, n = 3. P and S represent polymeric and soluble fractions of tubulin, respectively. Data are presented as mean ± SEM. ^**^P < 0.01, Student’s *t* test.

MAP4 is a key microtubule dynamics regulator in cancer cells. However, its function connection with ARID1A in CRC cells remains poorly understood. MAP4 activity is mainly regulated through its phosphorylation. MAP4 phosphorylation (p-MAP4) weakens its binding to microtubules, leading to microtubule depolymerization [15]. Therefore, to investigate the functional crosstalk between ARID1A and MAP4, we checked whether the MAP4 phosphorylation level is regulated by ARID1A. Immunoblot analysis revealed that p-MAP4 was upregulated by *ARID1A* knockout or silencing (Fig. 6A, E), and downregulated by ectopic overexpression of *ARID1A* (Fig. 6B, F). MAP4 phosphorylation levels were negatively correlated with ARID1A levels, suggesting that ARIDIA plays a role in MAP4 phosphorylation. MAP4 phosphorylation is regulated mainly by protein kinases. To identify kinases involved in MAP4 phosphorylation, we analyzed the transcriptome profiles of the HCT116 *ARID1A* isogenic cell pair. The top 20 KEGG enriched pathways are shown (Fig. S6A), among which the PI3K/AKT signaling pathway attracted our attention. Previous reports have shown that ARID1A loss tends to activate the PI3K/AKT signaling pathway in some cancers [30, 31]. Moreover, MAP4 interacts with PI3K directly through the C2 domain [32]. Given that PI3K/AKT signaling pathway was activated by *ARID1A* knockout or silencing (Figs. 6C, E and S6B), and inactivated by ectopic overexpression of *ARID1A* in CRC cells (Fig. 6D, F), PI3K could be a potential mediator of the regulatory relationship between ARID1A and p-MAP4. To test this hypothesis, we first confirmed the interaction between MAP4 and PI3K using Co-immunoprecipitation (Co-IP) and reciprocal Co-IP, followed by western blot analyses. These assays confirmed a stable interaction between MAP4 and PI3K (Figs. 6G–I and S6C–E). We further treated HCT116 *ARID1A* isogenic cells with PI3K inhibitor (LY294002), PKD1 inhibitor (GSK2334470) and AKT inhibitor (MK2206), respectively, and the phosphorylation status of MAP4 was examined. Inhibition of PI3K activity significantly downregulated p-MAP4 (Fig. 6J), whereas inhibition of PDK1 and AKT activity did not affect p-MAP4 expression (Fig. S6F, G). We also confirmed that PI3K inhibition reversed microtubule depolymerization induced by EMP treatment in *ARID1A* isogenic cells (Fig. 6K, L). These data suggest that PI3K, activated by ARID1A loss, interacts with MAP4 and causes MAP4 phosphorylation, thus enhancing EMP sensitivity in ARID1A-deficient cells.

To further examine whether the phosphorylation status of MAP4 have impact on microtubule destabilizers sensitization, cell viability was assessed after treating HCT116 and RKO *ARID1A* isogenic cell pairs with three microtubule destabilizers that act on different targets. Compared with HCT116 cells, microtubule destabilizers treatment, including vinorelbine (Fig. S7A, G), nocodazole (Fig. S7C, I), and colchicine (Fig. S7E, K), selectively inhibited the viability of ARID1A-deficient cells. Similar results were observed in the RKO *ARID1A* isogenic cell pair. Re-introduction of ARID1A in *ARID1A*-mutant RKO cells significantly reversed cell viability inhibition induced by vinorelbine (Fig. S7B, H), nocodazole (Fig. S7D, J) and colchicine (Fig. S7F, L) treatment. Above results illustrate that PI3K-mediated MAP4 phosphorylation enhances microtubule destabilizers sensitivity in ARID1A-deficient cells.

## Discussion

The development of safe and effective antitumor agents has been the focus of drug discovery. However, de novo drug development is characterized by high attrition rates, substantial costs and slow pace. Hence, the lower overall costs and shorter development timelines of drug repurposing has attracted the attention of researchers [33]. In the present study, we performed a large-scale drug screening in an approved drug library based on the principles of synthetic lethality and drug repurposing. We found that ARID1A-deficient cells were highly sensitive to EMP, and the synthetic lethal effect between EMP and ARID1A was MAP4-mediated microtubule dynamics dependent. The synthetic lethality of ARID1A and EMP was verified both in HCT116 *ARID1A* knockout cells and RKO *ARID1A* re-introduction cells. Phenotypically, EMP treatment selectively destabilized microtubule dynamics in ARID1A-deficient cells, resulting in defects in spindle assembly crucial for mitosis, consequently leading to abnormal cell division and eventually cell death. This phenotype is in agreement with EMP’s antimitotic properties; namely, EMP binds to microtubule-associated proteins, thereby inhibiting microtubule dynamics and leading to anaphase arrest [29, 34–36]. Inhibiting ARID1A expression is known to cause inhibition of cell division, which is potentially caused by the inhibition of microtubule formation [37]. Therefore, the combination of ARID1A deficiency and EMP treatment could cause the defects in spindle assembly and induce abnormal chromatin segregation, which are intolerable for cells and lead to cell apoptosis.

Mechanistically, ARID1A loss in CRC cells enhances MAP4 phosphorylation level, which promotes microtubule depolymerization, making cells vulnerable to antimicrotubule chemotherapy agents. Microtubules are highly dynamic filaments with dramatic structural rearrangements and length changes during the cell cycle, and factors that affect the spatial and temporal control of microtubule dynamics are often achieved through phosphorylation [25].

Phosphorylation is the most common type of posttranslational modification of MAPs, and this phosphorylation either dissociates them from the microtubule lattice or reduces their ability to stabilize microtubules. MAP4, as the target of EMP, is the most ubiquitously expressed MAPs, also regulating microtubule dynamics through its phosphorylation process [15, 38]. Under normal conditions, MAP4 binds to microtubules and maintains the stability of microtubules. Once phosphorylated, MAP4 dissociates from microtubules, leading to microtubule disassembly and dynamic instability [39]. In the present study, the phosphorylation level of MAP4 was negatively regulated by ARID1A, which indicates the potential contribution of ARID1A to the regulation of microtubule dynamics. Pharmacological or genetic perturbation of MAP4 recapitulated the synthetic lethality phenotype in ARID1A-deficient cells, further supporting the idea that MAP4 is a key microtubule dynamics regulator in ARID1A-deficient CRC, and it could serve as a therapeutic target.

Phosphorylation of MAPs are mainly regulated by protein kinases and phosphatase [15]. In our study, we identified that ARID1A loss upregulates MAP4 phosphorylation via PI3K activation. ARID1A loss simultaneously activated the PI3K/AKT pathway and upregulated p-MAP4, indicating a possible regulatory relationship between the PI3K/AKT pathway and MAP4 phosphorylation. Moreover, MAP4 phosphorylation in ARID1A-deficient cells was inhibited by PI3K inhibitor, but not PDK and AKT inhibitors, suggesting PI3K acts as a mediator in MAP4 phosphorylation induced by ARID1A loss. Furthermore, MAP4 interacts with PI3K and controls the distribution and localization of PI3K along the microtubules, thus affecting the PI3K/AKT signaling transduction [32], which further strengthens our findings. Our results verified that the interaction can not only affects PI3K/AKT cascades but also increases MAP4 phosphorylation level, leading to the impairment of MAP4’s ability to bind to and stabilize the microtubules. Phosphorylation of MAPs has a dramatic effect on microtubule dynamics, which in turn affects the efficacy of antimicrotubule agents, such as paclitaxel and vincristine. Inhibiting the phosphorylation of MAP4 increases microtubule stability, which enhances paclitaxel sensitivity in ovarian cancer cells [40]. Similarly, microtubule destabilization induced by glycogen synthase kinase-3β-mediated phosphorylation of Tau sensitizes human tumor cells to vincristine [41]. Our results indicate that, in the instance where PI3K is activated, selective diminishment of microtubule stability by MAP4 phosphorylation is achieved in ARID1A-deficient cells, increasing the response rate of EMP. This preferential induction of microtubule disruption was rescued by MAP4 overexpression or PI3K inhibition. Therefore, we have strong grounds to speculate that PI3K-mediated MAP4 phosphorylation of EMP-induced microtubule disruption only increases cell death in ARID1A-deficient CRC cells but not ARID1A-proficient cells, as the former is exposed to much lower doses of EMP. Therefore, identification the baseline of microtubule dynamics is crucial for individualized treatment with antimicrotubule agents in patients with cancer.

**Fig. 7:**
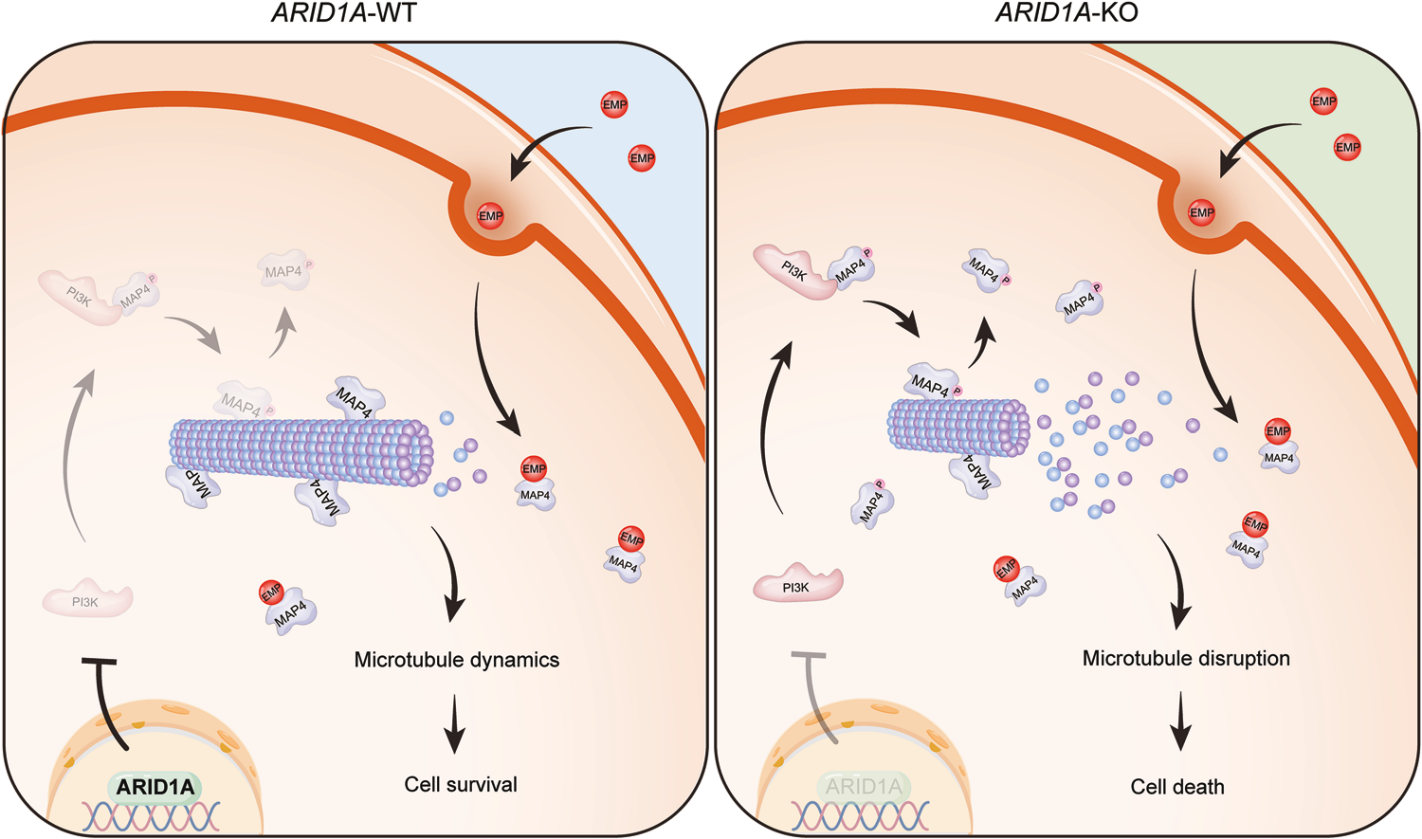
A working model illustrating the synthetic lethal effect between EMP and ARID1A. In ARID1A-proficient cells, ARID1A inhibits PI3K expression, resulting in a relatively low expression level of p-MAP4. MAP4 functions properly in regulating microtubule dynamics and stability. While in ARID1A-deficient cells, ARID1A loss leads to persistent activation of PI3K and promotes the phosphorylation of MAP4. When treated with EMP, a microtubule destabilizer, coupled with MAP4 phosphorylation, microtubule dynamics are severely disrupted, and eventually leading to mitotic cell death.

In summary, our findings revealed a novel synthetic lethality relationship between ARID1A and EMP, mediated through the regulation of microtubule dynamics by MAP4. ARID1A loss activates PI3K and promotes the interaction between PI3K and MAP4, which increases the phosphorylation level of MAP4, leading to microtubule instability and a reliance on the residual activity of MAP4. MAP4-targeting drug (EMP) treatment severely disrupts microtubule dynamics and affects bipolar spindle formation and positioning, ultimately inducing mitotic cell death in ARID1A-deficient cells (Fig. 7). As a tumor suppressor, ARID1A loss is correlated with tumor progression, with its frequency increasing with tumor-node-metastasis (TNM) stage: 7.4% in stage I, 24.1% in stage II, 22.2% in stage III, and 46.3% in stage IV. Our findings suggest that EMP could serve as a promising therapeutic option for CRC, particularly in advanced stages where ARID1A loss is more prevalent. Given that ARID1A mutations are commonly found across a wide range of malignancies, our results have broad implications for the development of targeted therapies through drug repurposing strategies.

## Supplementary information

Supplementary Material

Original Western Blot

## Data Availability

All supporting data are available within the article and Supplementary Files, or corresponding authors upon reasonable request.
