# Supplementary material for: MAP4 phosphorylation induced by ARID1A loss sensitizes colorectal cancer cells to EMP
Source: Cell Death Dis. 2025 Dec 8;17(1):83. doi: 10.1038/s41419-025-08286-5 (PMC12830715; doi:10.1038/s41419-025-08286-5)
Supplement: Supplementary file 2 — Original Western Blot [file 41419_2025_8286_MOESM2_ESM.pdf]

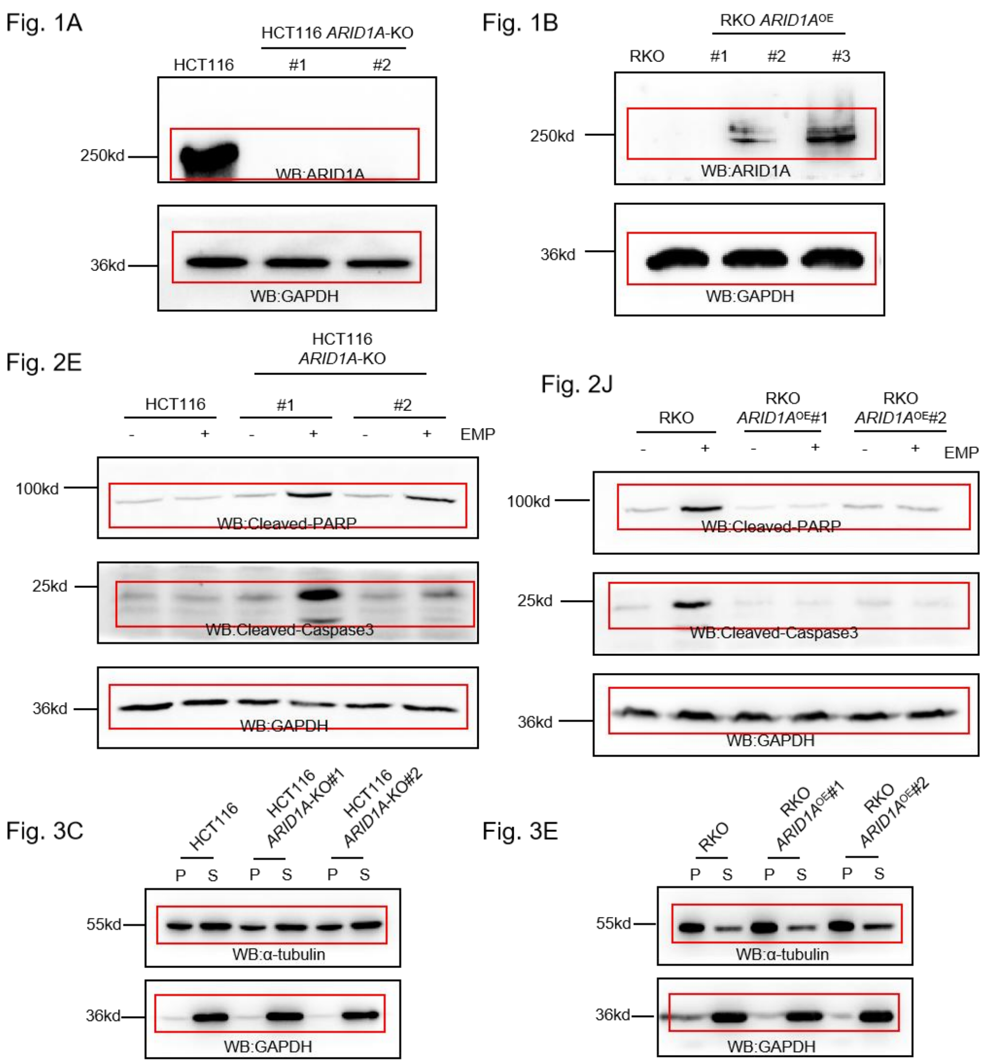

Original Western blots shown in Figures 1-3. Each figure corresponds to the western blots in the indicated Figure number.

Fig. 3I

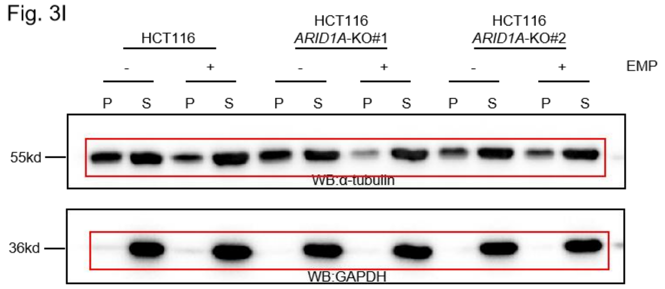

Fig. 3K

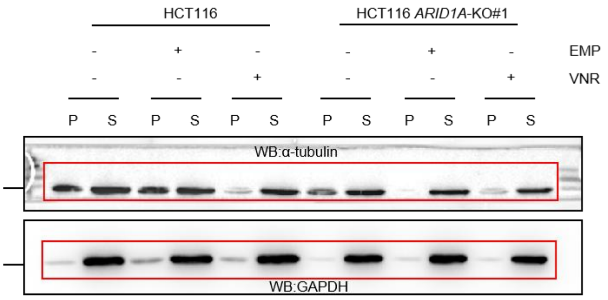

Original Western blots shown in Figure 3. Each figure corresponds to the western blots in the indicated Figure number.

Fig. 3M

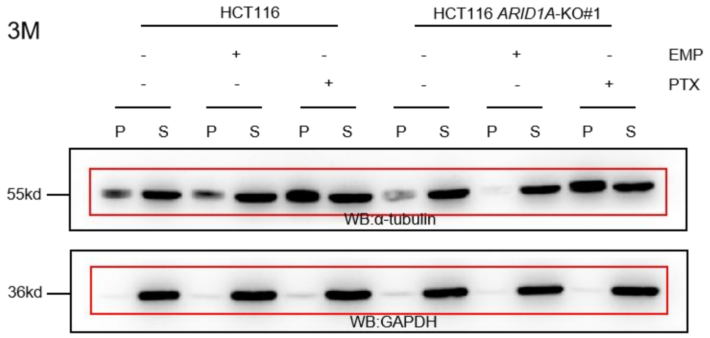

Fig. 3O

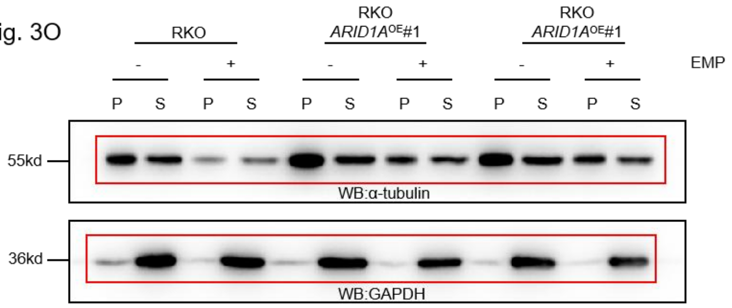

Original Western blots shown in Figure 3. Each figure corresponds to the western blots in the indicated Figure number.

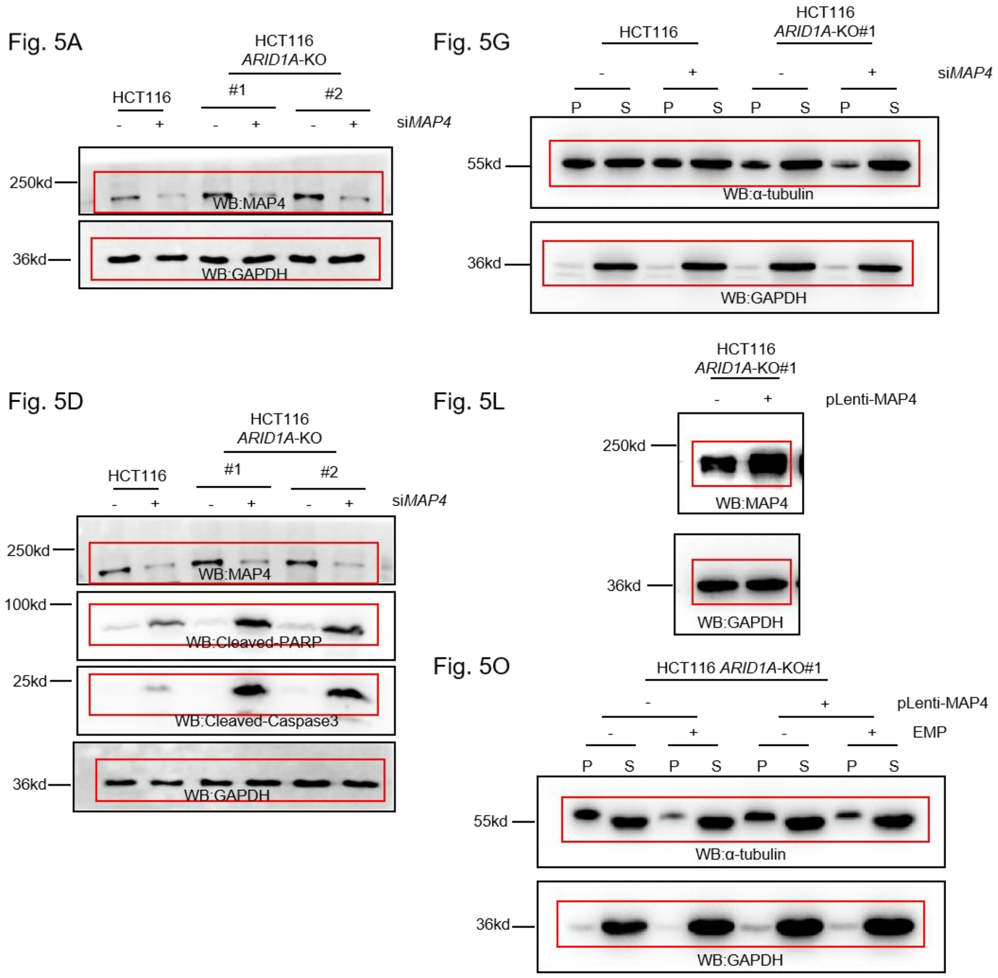

Original Western blots shown in Figure 5. Each figure corresponds to the western blots in the indicated Figure number.

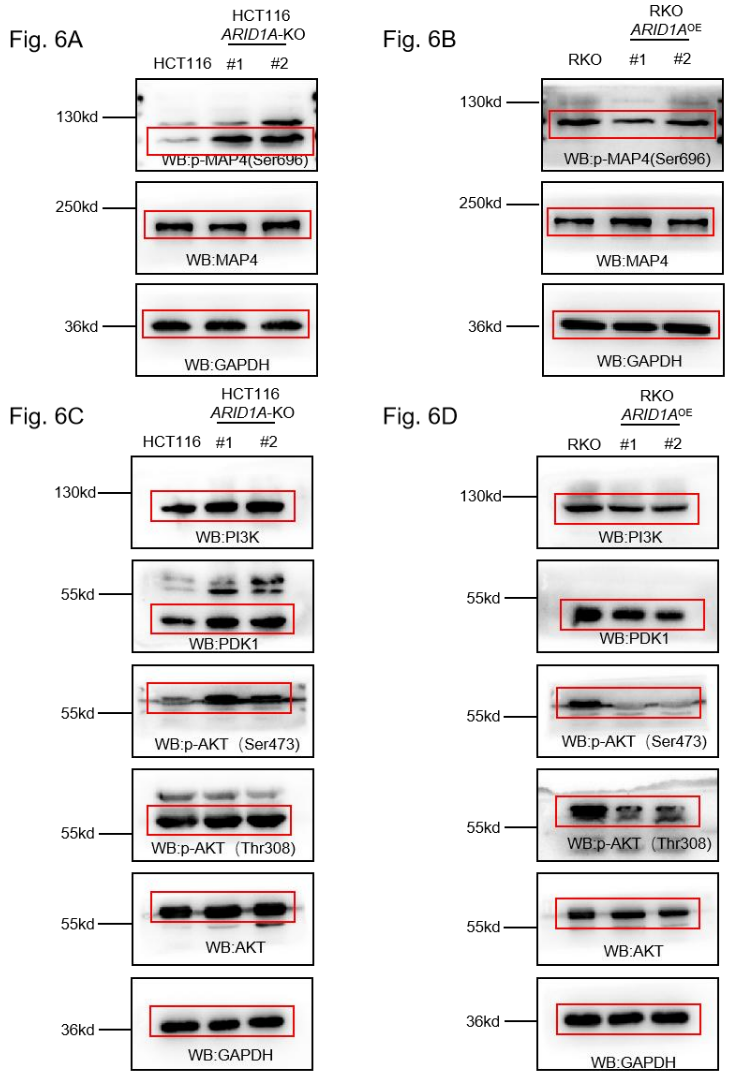

Original Western blots shown in Figure 6. Each figure corresponds to the western blots in the indicated Figure number.

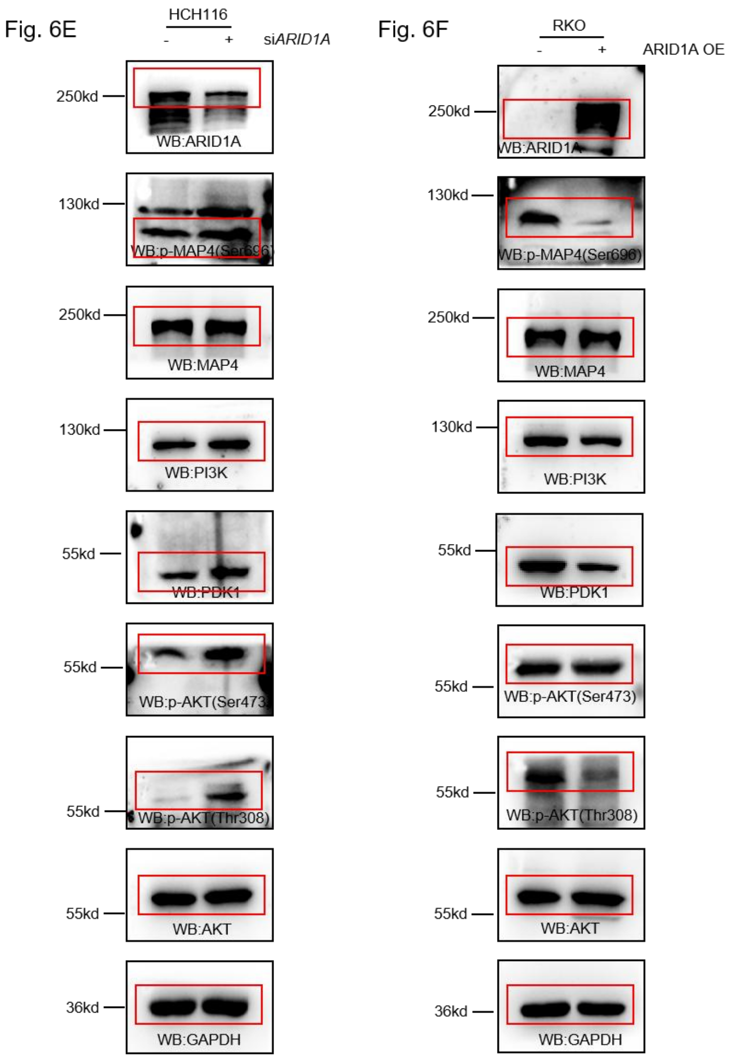

Original Western blots shown in Figure 6. Each figure corresponds to the western blots in the indicated Figure number.

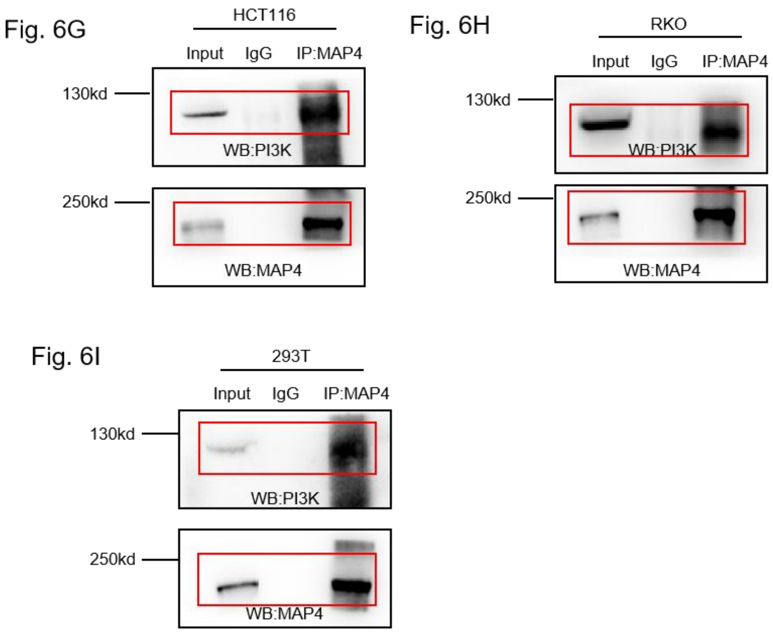

Original Western blots shown in Figure 6. Each figure corresponds to the western blots in the indicated Figure number.

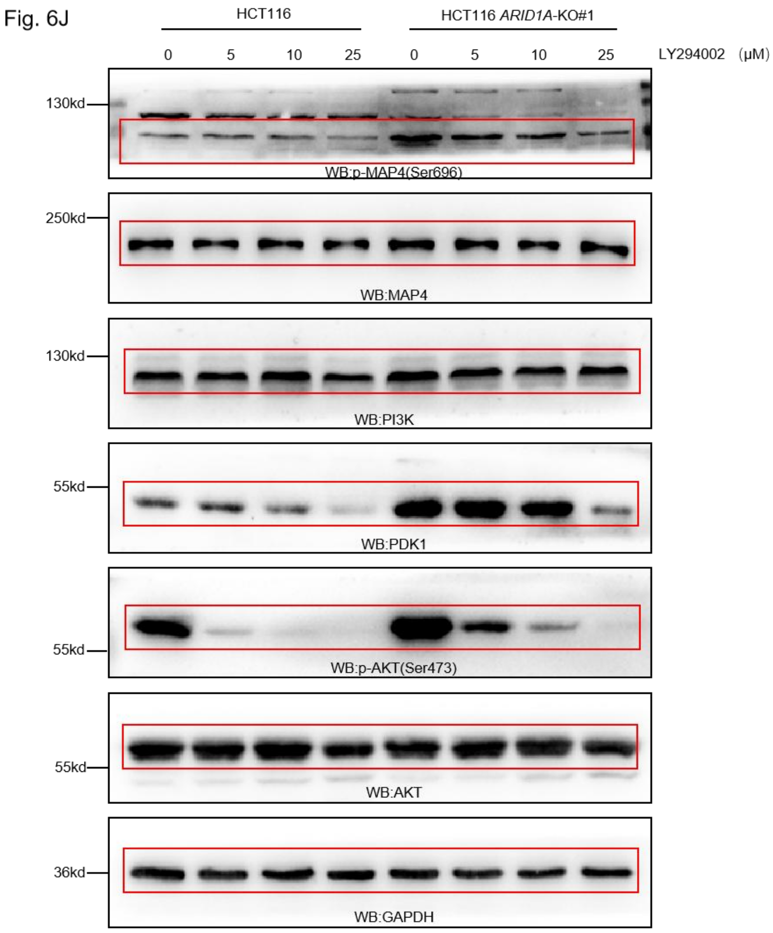

Original Western blots shown in Figure 6. Each figure corresponds to the western blots in the indicated Figure number.

Fig. 6K

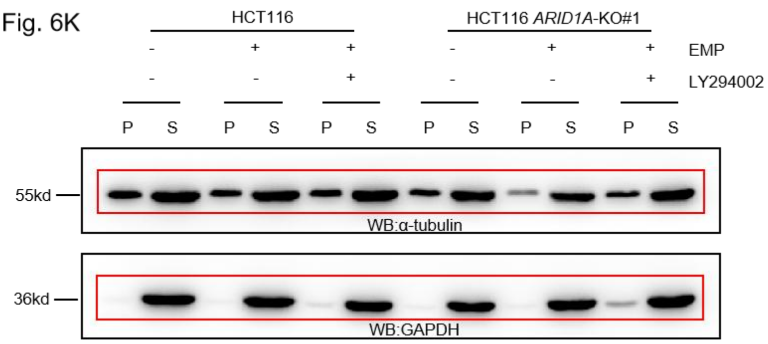

Fig. S2F

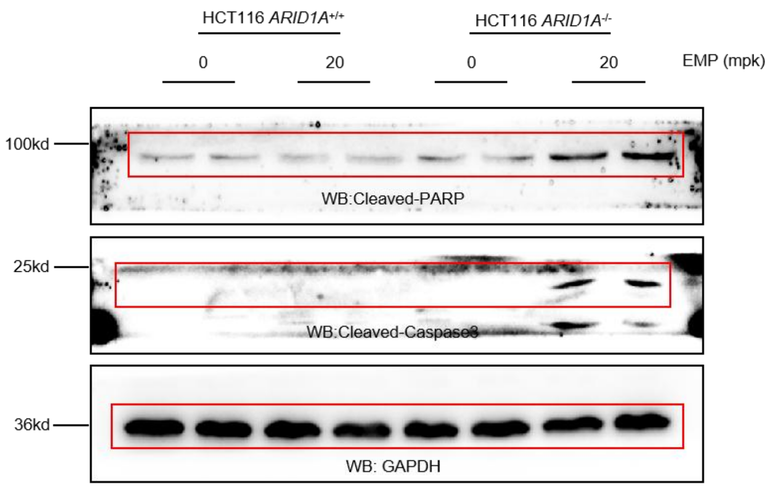

Original Western blots shown in Figure 6 and Supplementary Fig. S2. Each figure corresponds to the western blots in the indicated Figure number.

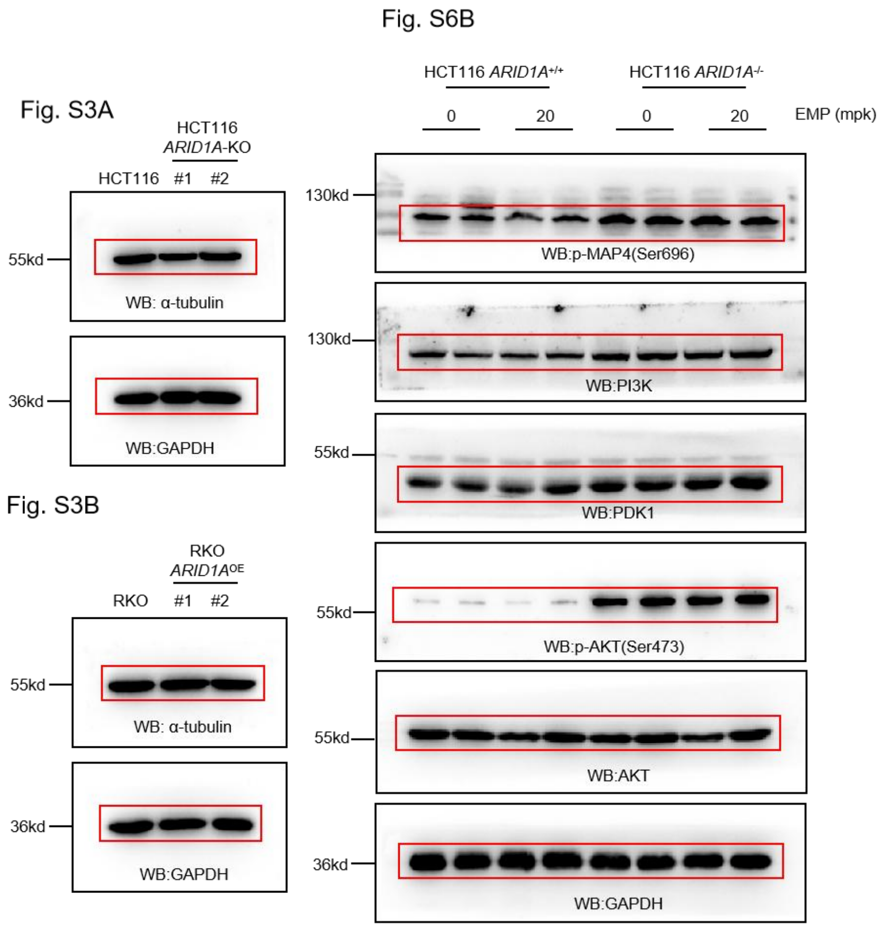

Original Western blots shown in Fig. S3 and S6. Each figure corresponds to the western blots in the indicated Figure number.

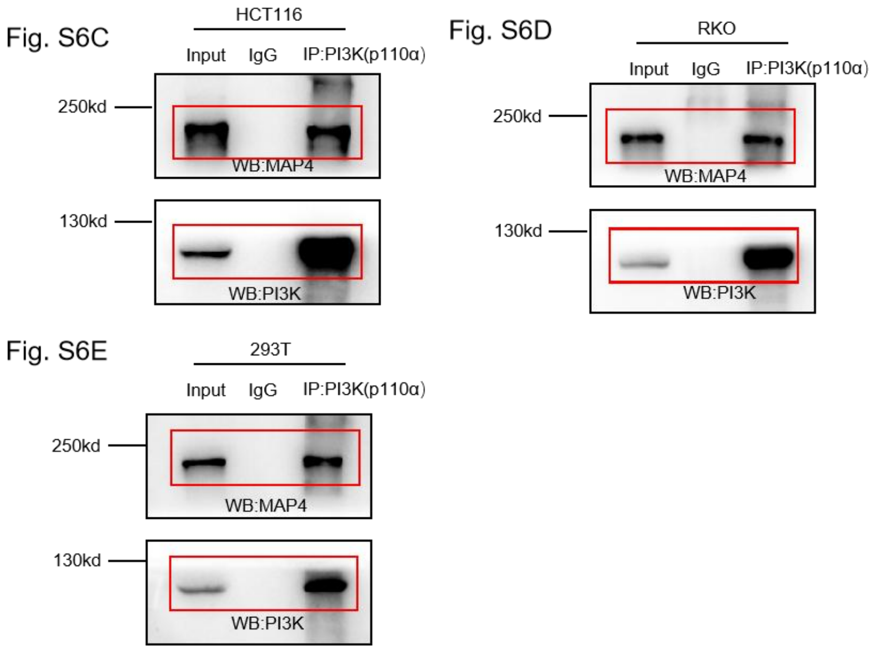

Original Western blots shown in Figure S6. Each figure corresponds to the western blots in the indicated Figure number.

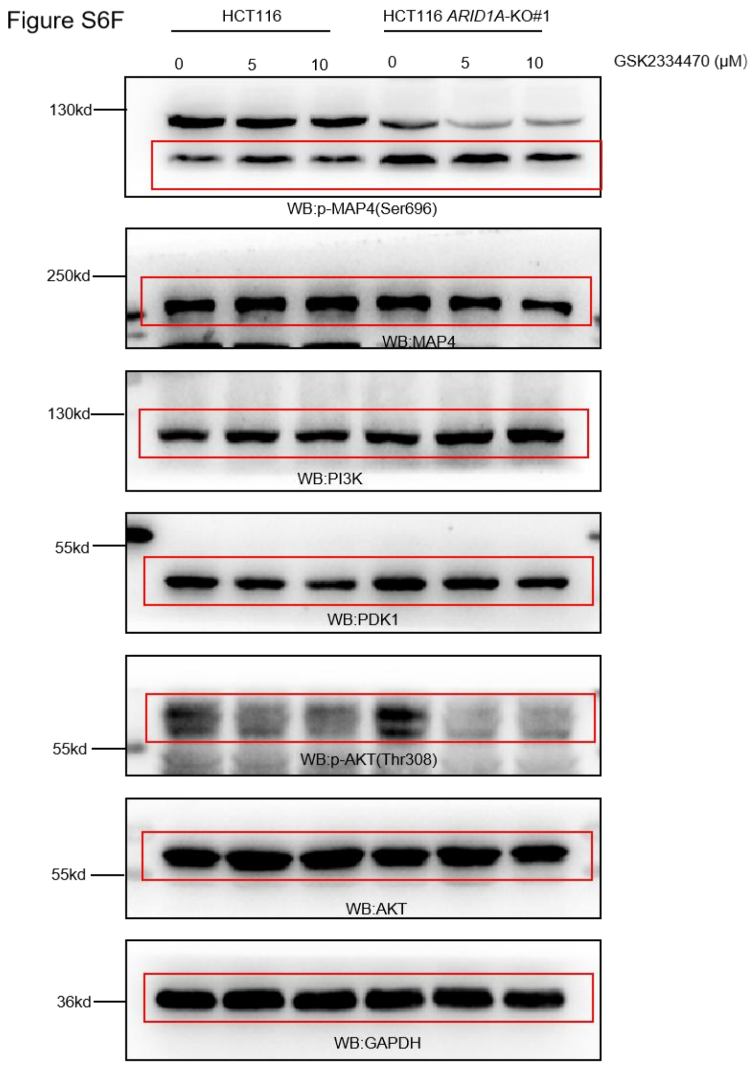

Original Western blots shown in Figure S6. Each figure corresponds to the western blots in the indicated Figure number.

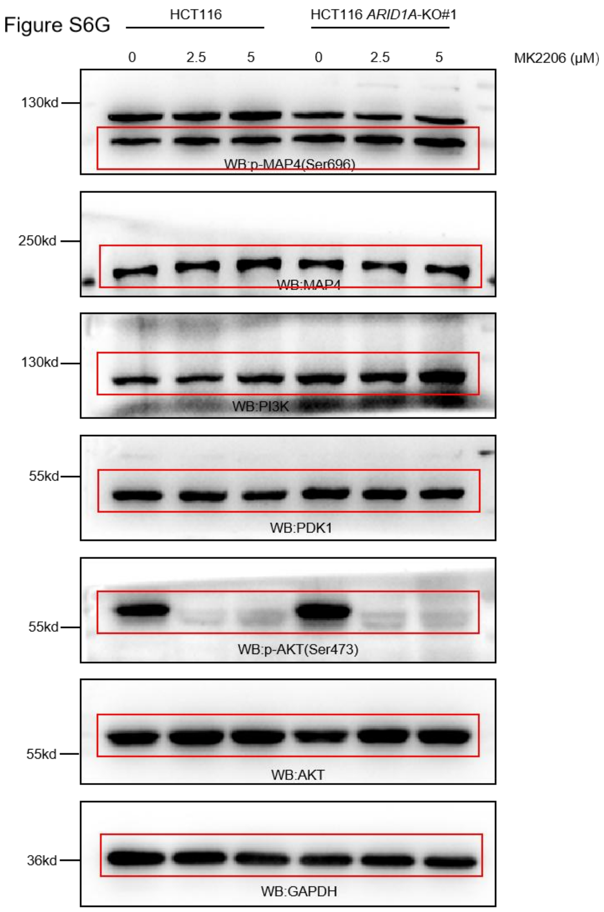

Original Western blots shown in Figure S6. Each figure corresponds to the western blots in the indicated Figure number.
