# Supplementary material for: MAP4 phosphorylation induced by ARID1A loss sensitizes colorectal cancer cells to EMP
Source: Cell Death Dis. 2025 Dec 8;17(1):83. doi: 10.1038/s41419-025-08286-5 (PMC12830715; doi:10.1038/s41419-025-08286-5)
Supplement: Supplementary file 1 — Supplementary Material [file 41419_2025_8286_MOESM1_ESM.doc]

Supplementary Material

**Fig. S1 Validation of synthetic lethality between ARID1A and candidate hits.** Dose-response curves of pyrvinium pamoate (**A**), afatinib dimaleate (**B**), belinostat (**C**), benzbromarone (**D**), bromocriptine mesylate (**E**), cisplatin (**F**), clofarabine (**G**), crisaborole (**H**), fosinopril sodium (**I**), lumacaftor (**J**), vildagliptin (**K**), n=3.

**Fig. S2 EMP is synthetic lethal with ARID1A**. (**A-D**) Colony formation of HCT116 (**A-B**) and RKO (**C-D**) *ARID1A* isogenic cell lines treated with indicated concentrations of EMP, n=3. (**E**) Representative image of HCT116 *ARID1A*^+/+^ and HCT116 *ARID1A*^-/-^ xenografts, n=5. (**F**) Apoptosis-related proteins in tumor masses isolated from mice were determined by western blotting. (**G**) Representative images of immunohistochemical staining of Ki 67 in tumor masses isolated from mice. (**H**) Body weight changes of mice treated with vehicle and 20 mgkg^-1^ EMP, n=5. In all relevant panels, data are presented as mean ± SD, ^*^*P*＜0.05, ^**^*P*＜0.01, Student’s *t* test.

**Fig. S3 ARID1A has a negligible influence on the expression level of total α-tubulin.** (**A-B**) Total α-tubulin expression levels in HCT116 (**A**) and RKO (**B**) *ARID1A* isogenic cell lines.

**Fig. S4 EMP disrupts mitotic spindles of ARID1A-deficient CRC cells**. (**A**) Representative images of spindle abnormalities. Scale bar, 5 μm. (**B**) Measurement of spindle length. All the bipolar and small-bipolar mitotic spindles were analyzed. (**C**) Analyses of abnormal spindle morphologies. All the mitotic spindles were analyzed and classified for quantitative analyses, n=3. (**D**) Analyses of mitotic phenotypes. All the metaphase spindles taken from fluorescence microscope were analyzed. It is notable that multiple mitotic defects phenotypes can co-occur within the same cell, n=3. (**E-F**) Cell cycle distribution (**E**) and cell population quantification (**F**) of RKO *ARID1A* isogenic cell pair treated with EMP are shown. Data are presented as mean ± SD.

**Fig. S5 MAP4 silencing induces mitotic defects in ARID1A-deficient cells**. (**A**) Representative images of mitotic defects. The white dotted lines indicate the cell boundaries, and the white arrow points the lagging chromosomes. Scale bars, 5 μm. (**B**) Analyses of mitotic phenotypes. All the metaphase spindles taken from confocal microscope were analyzed. It is notable that multiple mitotic defects phenotypes can co-occur within the same cell. Data are presented as mean ± SD. ^**^*P*＜0.01, Student’s *t* test.

**Fig. S6 PI3K interacts with MAP4 and promotes its phosphorylation**. (**A**) KEGG enrichment analyses of transcriptome sequencing. (**B**) Changes in protein expression level of phosphorylated MAP4 and PI3K/AKT signaling pathway in tumor masses isolated from mice. (**C-E**) Interaction between MAP4 and PI3K. Reciprocal coimmunoprecipitation assays were performed in HCT116 (**C**), RKO (**D**) and 293T (**E**) cell lines. (**F-G**) Changes of phosphorylated MAP4 expression level in HCT116 *ARID1A* isogenic cell pair treated with GSK2334470 (PDK1 inhibitor) (**F**) and MK2206 (AKT inhibitor) (**G**).

**Fig. S7 MAP4 phosphorylation enhances microtubule destabilizer sensitivity**. (**A-L**) Representative images and quantification of cell viability in HCT116 and RKO *ARID1A* isogenic cell pairs treated with indicated concentrations of VNR (**A-B, G-H**), nocodazole (**C-D, I-J**) and colchicine (**E-F, K-L**). Scale bars, 200 μm. In all relevant panels, data are presented as mean ± SD, ^*^*P*＜0.05, ^**^*P*＜0.01, Student’s *t* test.
